# Oligomeric Procyanidins Interfere with Glycolysis of Activated T Cells. A Novel Mechanism for Inhibition of T Cell Function

**DOI:** 10.3390/molecules201019014

**Published:** 2015-10-20

**Authors:** Masao Goto, Manabu Wakagi, Toshihiko Shoji, Yuko Takano-Ishikawa

**Affiliations:** 1 National Food Research Institute, National Agriculture and Food Research Organization, 2-1-12 Kannondai, Tsukuba, Ibaraki 305-8642, Japan; E-Mails: masaogot@affrc.go.jp (M.G.); mana5998bu@affrc.go.jp (M.W.); 2 Institute of Fruit Tree Science, National Agriculture and Food Research Organization, 2-1 Fujimoto, Tsukuba, Ibaraki 305-8605, Japan; E-Mail: tshoji@affrc.go.jp

**Keywords:** procyanidin, T cell, activation, proliferation, interleukin-2, glycolysis

## Abstract

Procyanidins, which are flavonoids that are found in a variety of plant species, reduce or prevent immune disorders, such as allergy and autoimmune diseases, through an unknown mechanism. In the present study, we investigated the effects of procyanidins on the T cell receptor (TCR)-mediated responses of CD4^+^ T cells *in vitro*. Apple procyanidins strongly suppressed the proliferation of splenic CD4^+^ T cells that were stimulated by an anti-CD3ε antibody, as well as splenocytes stimulated by antigen, but did not alter interleukin (IL)-2 secretion from these cells. Furthermore, we found that oligomeric procyanidins strongly suppressed, in a degree of polymerization dependent manner, the proliferation of activated CD4^+^ T cells, as well as their production of effector cytokines, including glycolysis associated-cytokines, without affecting IL-2 secretion. Additionally, we investigated the inhibitory effects of oligomeric procyanidins on the glycolytic activity of activated CD4^+^ T cells. We show that pentameric procyanidin suppressed L-lactate production and glucose uptake in activated CD4^+^ T cells. These results suggest that oligomeric procyanidins suppress the functions of activated CD4^+^ T cells by interfering with glycolysis.

## 1. Introduction

CD4^+^ T cells are distributed throughout the body, particularly in lymphatic organs such as the spleen and lymph nodes, and they play an important role in the adaptive immune system. When the body encounters exogenous antigens, antigen-presenting cells digest them into peptides and present these peptides, which are bound to MHC class II molecules, on their surface. When CD4^+^ T cells are stimulated with these peptides on antigen-presenting cells, they are activated and engage in rapid growth and robust proliferation, secrete cytokines, and differentiate into one of several lineages of T helper cells, which are effector subtypes. T cell receptor (TCR)-mediated signaling by specific antigens first promotes the expression of interleukin (IL)-2, which is the most important cytokine for the activation and proliferation of T cells. Effector functions, including cell proliferation, the production of various effector cytokines, and differentiation, are initiated after IL-2 production. It is believed that disorders in these processes lead to immune-related diseases, such as allergy and autoimmune diseases. Recent evidence has shown that each activation state requires distinct metabolic pathways. Mitochondrial metabolism, not glucose metabolism, is essential for IL-2 expression, although effector functions depend on glycolytic activity [1,2,3,4].

Procyanidins are a type of flavonoid that are widely distributed in a variety of plant species. Apples are one of the major dietary sources of procyanidins [5,6]. Procyanidins consist of (+)-catechin and (−)-epicatechin units that link together and have many isomeric forms with different chemical characteristics, depending on the extent of polymerization and the nature of the constituent units [7,8,9]. The content of polymeric forms depend both on the cultivar and cultivation management [10,11,12]. Procyanidins have medicinal benefits when used to treat diseases such as cardiovascular disease [13,14,15], inflammation [16,17], cancer, [18,19,20], allergies [21,22], infection [10], and autoimmune diseases [9,23]. A previous study indicated that oligomeric procyanidins prevent the aggravation of clinical symptoms and suppress antigen-specific immune responses in experimental autoimmune encephalomyelitis [9]. Therefore, procyanidins might modulate immune system functions, but the comprehensive mechanism of their pharmacologic effects on the immune system is unclear. In this work, we investigated the immune-modulating effects of apple derived procyanidins and oligomeric procyanidins to clarify the pharmacologic mechanism of procyanidins in the activation of primary CD4^+^ T cells.

## 2. Results

### 2.1. Apple Procyanidins (APCs) Suppress Splenic T Cell Proliferation without Reducing IL-2 Secretion

**Figure 1:**
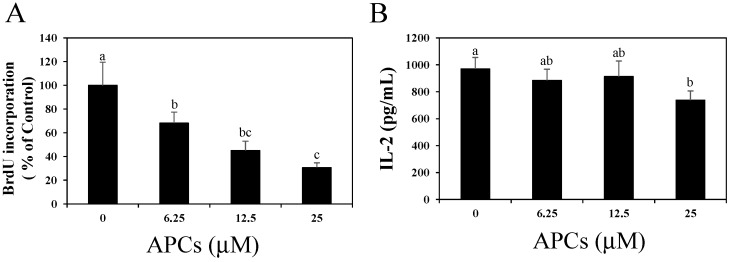
Dose-dependent effects of apple procyanidins (APCs) on antigen-specific cell proliferation (**A**) and interleukin (IL)-2 production (**B**) of activated splenocytes from naïve DO11.10 mice. Pooled splenocytes were stimulated with 7.5 μM of ovalbumin (OVA) in the presence of APCs (0–25 μM). (**A**) Splenocyte proliferation was evaluated by measuring the incorporation of BrdU after 72 h of stimulation. The group without APCs treatment (0 μM of APCs) was indicated as control. The data shown are the means ± SD from three independent experiments; (**B**) IL-2 secretion 48 h after stimulation was measured by ELISA. The data shown are the means ± SD from triplicate cultures. Values not sharing a common letter (a, b, c) differ significantly at *p* < 0.05 by the Tukey-Kramer multiple comparison test.

Splenocytes from DO11.10 mice were stimulated with 7.5 μM of chicken egg ovalbumin (OVA) in the presence of APCs (0, 6.25, 12.5, or 25 μM). The co-addition of APCs during antigen-specific activation resulted in a dose-dependent reduction in BrdU incorporation. The incorporation was significantly reduced at APC concentrations of 6.25 μM and higher (Figure 1A), although antigen-specific IL-2 secretion from splenocytes was not altered, except in the presence of 25 μM of APCs (Figure 1B).

**Figure 2:**
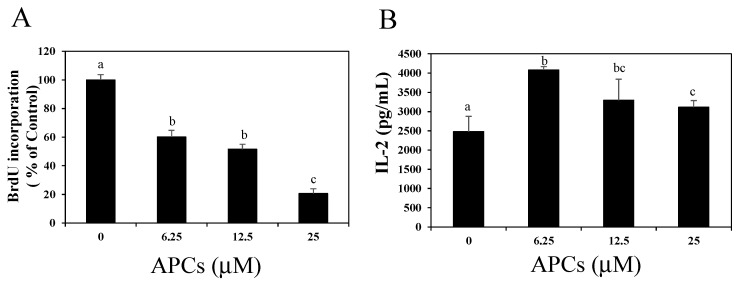
Dose-dependent effects of apple procyanidins (APCs) on cell proliferation (**A**) and interleukin (IL)-2 production (**B**) of activated CD4^+^ T cells. Splenic CD4^+^ T cells were stimulated with an anti-CD3ε monoclonal antibody in the presence of APCs (0–25 μM). (**A**) Cell proliferation was evaluated by measuring the incorporation of BrdU after 72 h of stimulation. The group without APCs treatment (0 μM of APCs) was indicated as control. The data shown are the means ± SD from three independent experiments; (**B**) IL-2 secretion 48 h after stimulation was measured by ELISA. The data shown are the means ± SD from triplicate cultures. Values not sharing a common letter (a, b, c) differ significantly at *p* < 0.05 by the Tukey-Kramer multiple comparison test.

Because CD4^+^ T cells greatly contribute to antigen-specific cell proliferation and IL-2 production from the splenocytes of DO11.10 mice, we investigated the effects of APCs on activated CD4^+^ T cells. CD4^+^ T cells separated from the splenocytes of DO11.10 mice were activated following TCR stimulation by an anti-CD3ε monoclonal antibody (mAb) in the presence of APCs (0, 6.25, 12.5, or 25 μM). The co-addition of APCs resulted in a dose-dependent reduction in BrdU incorporation, as was also the case when splenocytes were activated by the specific antigen (Figure 2A). Additionally, IL-2 secretion from splenic CD4^+^ T cells was significantly augmented by 6.25 μM and higher concentrations of APCs (Figure 2B).

We did not observe any acute toxicity to the cells under our experimental conditions (Figure S1).

### 2.2. Oligomeric Procyanidins Suppress T Cell Proliferation without Reducing IL-2 Secretion

**Figure 3:**
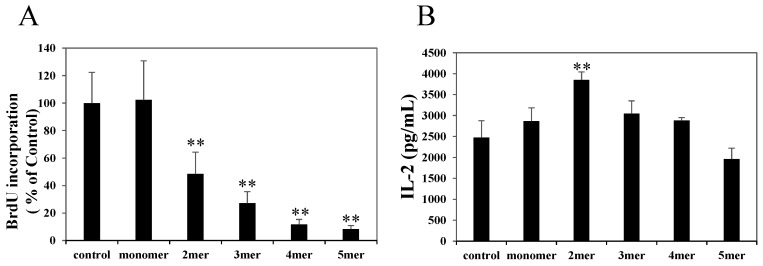
Effects of oligomeric procyanidins on cell proliferation (**A**) and interleukin (IL)-2 production (**B**) of activated CD4^+^ T cells. Splenic CD4^+^ T cells were stimulated with an anti-CD3ε monoclonal antibody in the presence of oligomeric procyanidins (25 μM). (**A**) Cell proliferation was evaluated by measuring the BrdU incorporation after 72 h of stimulation. The data shown are the means ± SD from three independent experiments; (**B**) IL-2 secretion 48 h after stimulation was measured by ELISA. The data shown are the means ± SD from triplicate cultures. Statistical comparisons were performed using analysis of variance with Dunnett’s multiple comparison of means test. Significance is relative to a negative control (** *p* < 0.01)

Splenic CD4^+^ T cells were stimulated with an anti-CD3ε mAb in the presence of monomeric, dimeric, trimeric, tetrameric, or pentameric procyanidins (0, 6.25, 12.5, or 25 μM). These procyanidins, except for the monomeric one at a concentration of 25 μM, inhibited cell proliferation significantly compared with negative control (Figure 3A), and their inhibitory potencies depended on their degree of polymerization. These procyanidins inhibited cell proliferation in a dose-dependent manner (Figure S2). In contrast, these procyanidins, except for dimeric procyanidin, had no effect on IL-2 secretion from activated CD4^+^ T cells (Figure 3B).

### 2.3. Oligomeric Procyanidins Reduce Effector Cytokine Secretion

**Figure 4:**
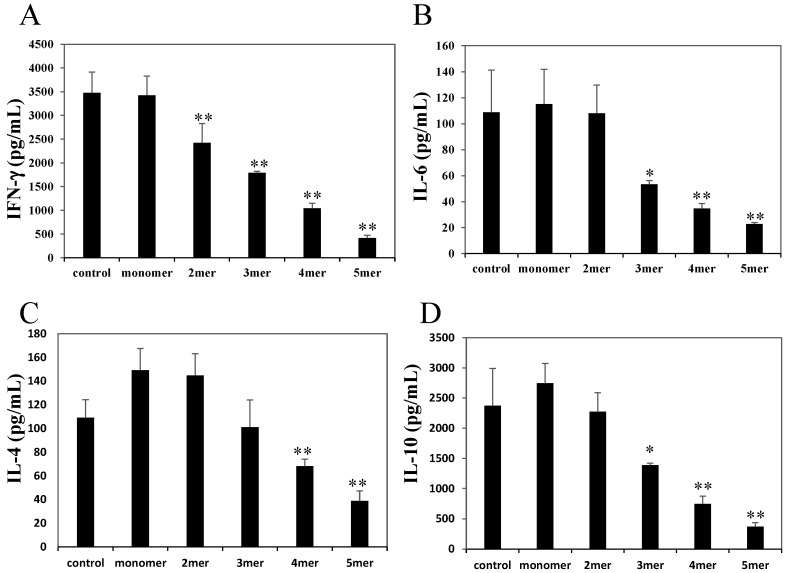
Effects of oligomeric procyanidins on the production of the effector cytokines, interferon (IFN)-γ (**A**), interleukin (IL)-6 (**B**); IL-4 (**C**); and IL-10 (**D**) by activated CD4^+^ T cells. Splenic CD4^+^ T cells were stimulated with an anti-CD3ε monoclonal antibody in the presence of oligomeric procyanidins (25 μM). The data shown are the means ± SD from triplicate cultures. Statistical comparisons were performed using analysis of variance with Dunnett’s multiple comparison of means test. Significance is relative to a negative control (* *p* < 0.05, ** *p* < 0.01).

We measured effector cytokine concentrations in the supernatants of splenic CD4^+^ T cells stimulated with an anti-CD3ε mAb in the presence of each procyanidin (0, 6.25, 12.5, or 25 μM). These procyanidins, except for the monomeric one at a concentration of 25 μM, significantly inhibited interferon (IFN)-γ secretion compared with vehicle (Figure 4A). Trimeric and higher oligomeric procyanidins at a 25 μM concentration significantly inhibited IL-6 secretion (Figure 4B). IL-4 and IL-10 secretion was inhibited by tetrameric and pentameric procyanidins (Figure 4C,D).

The inhibition occurred in a dose-dependent manner (Figure S3). Inhibitory potencies seemed to depend on the degree of polymerization. Procyanidins suppressed effector cytokines secreted from splenocytes stimulated by OVA in a similar manner (Figure S4).

### 2.4. Pentameric Procyanidin Reduces the Glycolytic Activity of Activated CD4^+^ T Cells

**Figure 5:**
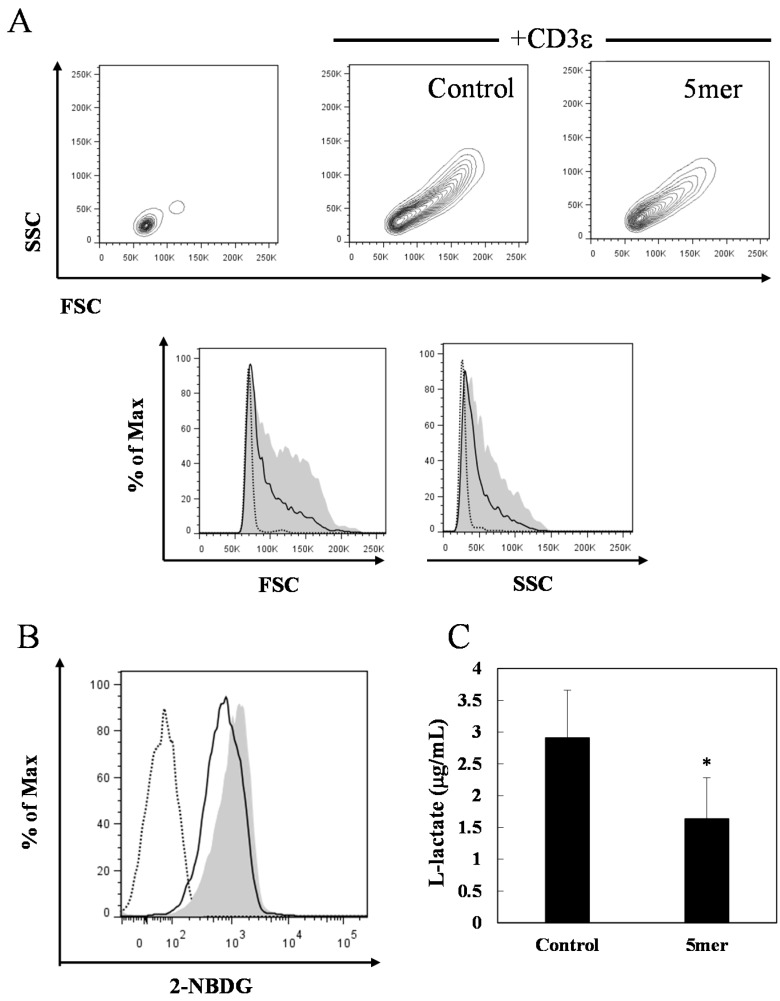
Effects of pentameric procyanidin on the glycolytic activities of activated CD4^+^ T cells. Cell growth (**A**), glucose uptake (**B**); and l-lactate production (**C**) of splenic CD4^+^ T cells stimulated with an anti-CD3ε monoclonal antibody (mAb) in the presence of pentameric procyanidin (25 μM) for 24 h. (**A**) The dotted line indicates the signal from cells that were not stimulated with the anti-CD3ε mAb. The shaded plot and the black line represent the signals from cells cultured with 0 and 25 μM of pentameric procyanidin, respectively; (**B**) The dotted line indicates the signal from cells incubated in the absence of 2-(*N*-[7-nitrobenz-2-oxa-1,3-diazol-4-yl]amino)-2-deoxyglucose (2-NBDG). The shaded plot and the black line represent the signals from cells cultured with 0 and 25 μM of pentameric procyanidin, respectively; (**C**) The data shown are the means ± SD from quintuplicate cultures. Statistical comparisons were performed using the Student’s *t*-test. Significance is relative to the negative control (* *p* < 0.05). FSC, forward scatter; SSC, side scatter.

Because IFN-γ [3,24] and IL-6 [25,26] are known to be associated with the glycolytic activity of T cells, we estimated the effects of oligomeric procyanidin on the glycolytic activity of activated CD4^+^ T cells. We measured glucose uptake and l-lactate production in activated CD4^+^ T cells in the presence of pentameric procyanidin. Pentameric procyanidin at a concentration of 25 μM decreased glucose uptake (Figure 5B) and inhibited l-lactate production significantly (Figure 5C) compared with negative control. Flow cytometric analyses of the forward scatter (FSC; an indicator of cell size) and side scatter (SSC: a measure of cell structural complexity) profiles were used to estimate the growth of activated T cells. Pentameric procyanidin supplementation reduced the number of large granular cells (Figure 5A).

## 3. Discussion

In the present study, we investigated the effects of procyanidins on activated primary CD4^+^ T cells. Although APCs strongly suppressed antigen-specific proliferation in the primary splenocytes of DO11.10 mice, they only slightly suppressed IL-2 secretion (Figure 1). APCs also suppressed the proliferation of splenic CD4^+^ T cells stimulated with an anti-CD3ε mAb without affecting IL-2 secretion (Figure 2). We also showed that oligomeric procyanidins derived from APCs did not suppress IL-2 secretion (Figure 3B), although they strongly inhibited proliferation (Figure 3A) and effector cytokine secretion (Figure 4) in splenic CD4^+^ T cells stimulated with an anti-CD3ε mAb. Moreover, their inhibitory potencies depended on their degree of polymerization (Figure 3A and Figure 4). This is the first report to show that APCs and their components suppress the functions of activated T cells without interfering with IL-2 secretion.

IL-2 is produced during the early stage of T cell activation, and it is crucial for T cell proliferation. Various immunosuppressive compounds, such as flavonoids [27,28,29], FK506, and cyclosporine A [30], suppress the proliferation of activated T cells by interfering with IL-2 production. However, in the present study, APCs and their components did not suppress IL-2 secretion, whereas they inhibited the proliferation of activated T cells, as well as their secretion of effector cytokines. Therefore, our results, as well as those of the aforementioned studies, suggest that oligomeric procyanidins suppress the functions of activated T cells in a different manner than other immunosuppressive compounds. Evidence is now emerging that each T cell function requires distinct metabolic pathways during a variety of activation states [1,2,3,4]. IL-2 production in activated T cells depends on reactive oxygen species (ROS) that are produced by mitochondrial metabolism, and the subsequent cell proliferation depends on glycolysis [2]. Some researchers speculated that antioxidants might interfere with ROS signaling and inhibit IL-2 expression [2,31,32,33]. In contrast, rapamycin, an immunosuppressive macrolide, does not affect IL-2 production, although it inhibits cell proliferation [30,34]. It has been reported that rapamycin inhibits glycolytic activity [35,36,37] by interfering with the mechanistic target of rapamycin (mTOR)-related signaling pathway, which upregulates the enzymes necessary for glycolysis [38]. Therefore, we speculated that, as with rapamycin, oligomeric procyanidins inhibit T cell proliferation by interfering with glycolysis. Recent studies have shown that blocking glycolysis induces marked inhibitions of IL-6 [25,26] and IFN-γ secretion [3,24]. Indeed, our results showed that oligomeric procyanidins suppressed both IFN-γ and IL-6 secretion by activated T cells (Figure 4A,B). The level of extracellular l-lactate correlates proportionally with intracellular glycolytic activity [39,40], and we showed that pentameric procyanidin decreased the level of extracellular l-lactate in activated T cells (Figure 5C). Within the first 24 h after TCR stimulation, T cells drive glycolysis to provide metabolites for macromolecule biosynthesis, and they increase their size and intracellular structural complexity [41,42]. We showed that pentameric procyanidin reduced the population of large granular activated CD4^+^ T cells (Figure 5A). Glucose uptake increases when glycolysis is upregulated [41,43], and we showed that pentameric procyanidin suppressed glucose uptake by activated T cells (Figure 5B). These results indicate that oligomeric procyanidins interfere with glycolysis and inhibit the functions of activated T cells.

In the present study, we suggested that the magnitude of the suppressive effects of oligomeric procyanidins seemed to be proportional to their degree of polymerization. It has been reported that the hydrophilicity of trimeric and higher oligomeric procyanidins impairs their transport into cells [9,44]. However, some reports suggest that oligomeric procyanidins affect the interaction of ligands with their receptors on the cell surface [16,45,46], and one of these receptors was detected on the surface of CD4^+^ T cells [47]. Oligomeric procyanidins could be agonists of these receptors. From these reports, we assume that oligomeric procyanidins might suppress T cell activation by binding their receptors. Therefore, it is necessary to investigate the interaction of oligomeric procyanidins with their receptors in CD4^+^ T cells.

Recently, it was indicated that cultivar and cultivation management can modify the anti-*Candida* activity and the content of polymeric forms of grape seeds procyanidins [10]. Regarding apples, the profile of procyanidins is different from each cultivar [11,12]. Therefore it is necessary to further investigate the effects of cultivar on immune-modulating effects of APCs.

## 4. Experimental Section

### 4.1. Reagents

APCs, with a mean degree of polymerization of approximately four, and procyanidins, from monomers to pentamers, were prepared from apples (*Malus pumila* cv. Fuji) using preparative column chromatography as described by a previous study [48]. Briefly, APCs were prepared from apple juice using a Sepabeads SP-850 (Mitsubishi Kasei Co., Ltd., Tokyo, Japan) preparative column with aromatic synthetic adsorbents. Apple polyphenol extracts were lyophilized, and the powder obtained was dissolved in distilled water and adjusted to pH 6.5 with 5 M NaOH. The sample was applied to a Diaion HP-20ss (Mitsubishi Kasei Co., Ltd.) column, and after rinsing the column with distilled water, the procyanidin fraction was eluted with 25% ethanol. Finally, the eluate was concentrated by rotary evaporation at 45 °C and lyophilized as the APC fraction. The APC fraction comprised dimers (25.1%), trimers (8.7%), tetramers (9.5%), pentamers (9.0%), hexamers (4.7%), heptamers (3.1%), and octamers or higher polymers (15.4%), and also included flavan-3-ol monomers (17.2%). Furthermore, procyanidins, from monomers to pentamers, were prepared by preparative normal-phase chromatography [48]. APCs and procyanidins were dissolved in sterile Dulbecco’s phosphate-buffered saline (D-PBS, Wako Pure Chemicals, Osaka, Japan) at a concentration of 5–10 mg/mL and were stored at −30 °C as stock solutions. The stock solutions were diluted with culture medium immediately before use.

OVA grade V was purchased from Sigma-Aldrich (St. Louis, MO, USA). Anti-mouse CD3ε mAb clone 145-2C11 and 7-Aminoactinomycin D (7-AAD) were purchased from eBioscience Inc. (San Diego, CA, USA).

### 4.2. Preparation of Mice Splenocytes and Splenic CD4^+^ T Cells

DO11.10 mice express an αβ TCR that is specific for the OVA peptide 323–339, which is MHC class II I-A^d^ restricted, and their naïve CD4^+^ T cells can respond to the OVA peptide without prior sensitization. DO11.10 mice were purchased from the Jackson Laboratory (Boston, MA, USA) and maintained in our specific pathogen-free animal facilities. Animals were fed a standard diet of rodent chow and water *ad libitum*. Animal studies were reviewed and approved by the Animal Care and Use Committee of the National Food Research Institute, the National Agriculture and Food Research Organization (NARO), Japan.

Splenocytes were prepared and pooled from 3–4 female DO11.10 mice (8–12 weeks of age) by mechanical dispersion, and CD4^+^ T cells were separated from pooled splenocytes with CD4 (L3T4) MicroBeads, mouse (Miltenyi Biotec, Bergisch Gladbach, Germany) according to the manufacturer’s instructions.

### 4.3. Cell Cultures and Treatment

Splenocytes or splenic CD4^+^ T cells were cultured in RPMI-1640 (Sigma-Aldrich) containing 2-mercaptoethanol (50 μM), penicillin (100 U/mL), streptomycin (100 μg/mL), and 10% fetal calf serum (Biowest, Nuaille, France or PAA Laboratories, Austria) in culture plates (Nunc, Boston, MA, USA). Splenocytes were stimulated with 7.5 μM of OVA peptide, and CD4^+^ T cells were stimulated with plate-bound anti-CD3ε mAb (0.5 μg/mL) in the presence of APCs or procyanidins.

### 4.4. Measurement of Cell Proliferation

To assess cell proliferation, 1 × 10^5^ splenocytes or splenic CD4^+^ T cells were cultured in a total volume of 100 μL per well in 96-well plates with duplicate wells per treatment. Cell proliferation was estimated 72 h after stimulation with a BrdU chemiluminescent cell-proliferation ELISA kit (Roche Molecular Biochemicals, Basel, Switzerland) according to the manufacturer’s instructions. To estimate the effects of APCs or procyanidins on cell proliferation, BrdU uptake in the absence of reagents was normalized to 100%.

### 4.5. Cytokine Measurements

To measure cytokines in culture supernatants, splenocytes were cultured at 3 × 10^5^ cells/well in a total volume of 300 μL per well, and splenic CD4^+^ T cells were cultured at 1 × 10^5^ cells/well in a total volume of 100 μL per well. Cell cultures were conducted in 96-well plates with triplicate wells per treatment. Culture supernatants were collected 48 h (for IL-2 and IL-4 measurements) and 72 h (for IL-6, IL-10, and IFN-γ measurements) after stimulation. Supernatants were stored at −30 °C until used. Cytokines were measured using Mouse Cytokine ELISA Ready-SET-Go! kits (eBioscience) according to the manufacturer’s instructions.

### 4.6. Measurement of l-Lactate

To measure l-lactate in the culture supernatants, splenic CD4^+^ T cells were cultured at 2 × 10^5^ cells/well in a total volume of 100 μL per well in 96-well plates with quintuplicate wells per treatment. Culture supernatants were collected 24 h after stimulation. Supernatants were stored at −30 °C until used. l-lactate was measured using the Glycolysis Cell-Based Assay Kit (Cayman Chemical, Ann Arbor, MI, USA) according to the manufacturer’s instructions.

### 4.7. Measurement of Glucose Uptake

To assess glucose uptake, splenic CD4^+^ T cells were cultured at 5 × 10^5^ cells/well in a 48-well plate. Cells were collected 24 h after stimulation and incubated with 100 μM of the fluorescently-labeled glucose analog 2-NBDG (Cayman Chemical) in glucose-free RPMI1640 (Wako Pure Chemicals) at 37 °C for 10 min, followed by staining with 7-AAD to eliminate dead cells. Samples were quantified on a FACS Canto II (BD Biosciences, San Jose, CA, USA), and the data were analyzed with FlowJo software version 7.6.5 (Tree Star, Inc., Ashland, OR, USA). In total, 5 × 10^3^ cells events were counted per sample.

### 4.8. Statistical Analysis

Results are presented as the mean ± standard deviation (SD). Statistical analysis was performed using the Tukey–Kramer multiple comparison test (Figure 1 and Figure 2; *p* values < 0.05 were considered to be statistically significant), analysis of variance (ANOVA) with Dunnett’s multiple comparison of means test (Figure 3 and Figure 4; * *p* values < 0.05 and ** *p* values < 0.01), and the Student’s *t* test (Figure 5C; *p* values < 0.05 were considered to be statistically significant).

## 5. Conclusions

According to experimental results, oligomeric procyanidins inhibit T cell functions by inhibiting glycolysis, possibly via signaling from cell membrane receptors. Thus, procyanidins might prevent immune disorders in a different manner than other immunosuppressive compounds.

## Supplementary Materials

Supplementary materials can be accessed at: http://www.mdpi.com/1420-3049/20/10/19014/s1.
