# Supplementary material for: Oligomeric Procyanidins Interfere with Glycolysis of Activated T Cells. A Novel Mechanism for Inhibition of T Cell Function
Source: Molecules. 2015 Oct 20;20(10):19014–26. doi: 10.3390/molecules201019014 (PMC6332502; doi:10.3390/molecules201019014)
Supplement: Supplementary file 1 [file molecules-20-19014-s001.pdf]

Supplementary Materials

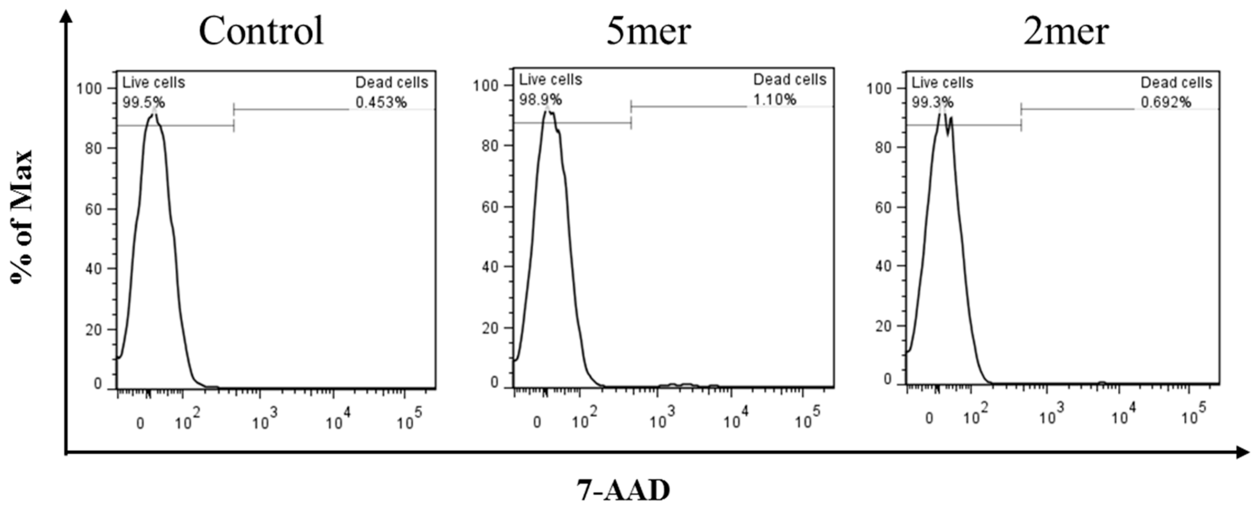

Figure S1. Toxicity of pentameric or dimeric procyanidin on the activated CD4$^+$ T cells. To assess toxicity of procyanidins, splenic CD4$^+$ T cells stimulated with an anti-CD3ε monoclonal antibody (mAb) in the presence of pentameric or dimeric procyanidin (25 μM) for 24 h, followed by staining with 7-Aminoactinomycin D (7-AAD). The group without oligomeric procyanidins treatment was indicated as control.

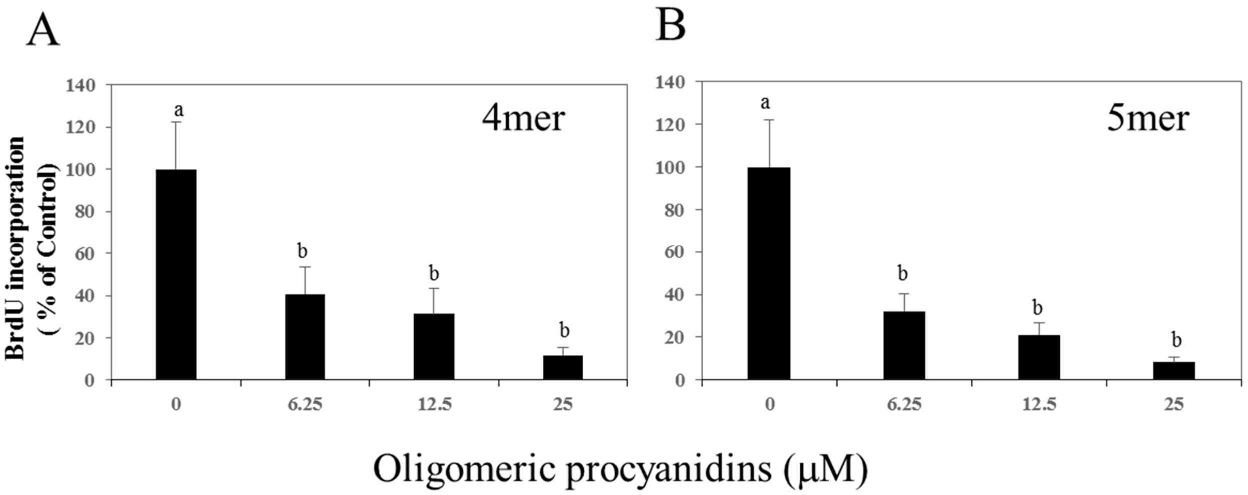

Figure S2. Dose-dependent effects of oligomeric procyanidins on cell proliferation of activated CD4$^+$ T cells. Splenic CD4$^+$ T cells were stimulated with an anti-CD3ε monoclonal antibody in the presence of tetrameric (**A**) or pentameric (**B**) procyanidins (0–25 μM). Cell proliferation was evaluated by measuring the BrdU incorporation after 72 h of stimulation. The group without oligomeric procyanidins treatment (0 μM) was indicated as control. The data shown are the means ± SD from three independent experiments. Values not sharing a common letter (a, b) differ significantly at $p < 0.05$ by the Tukey-Kramer multiple comparison test.

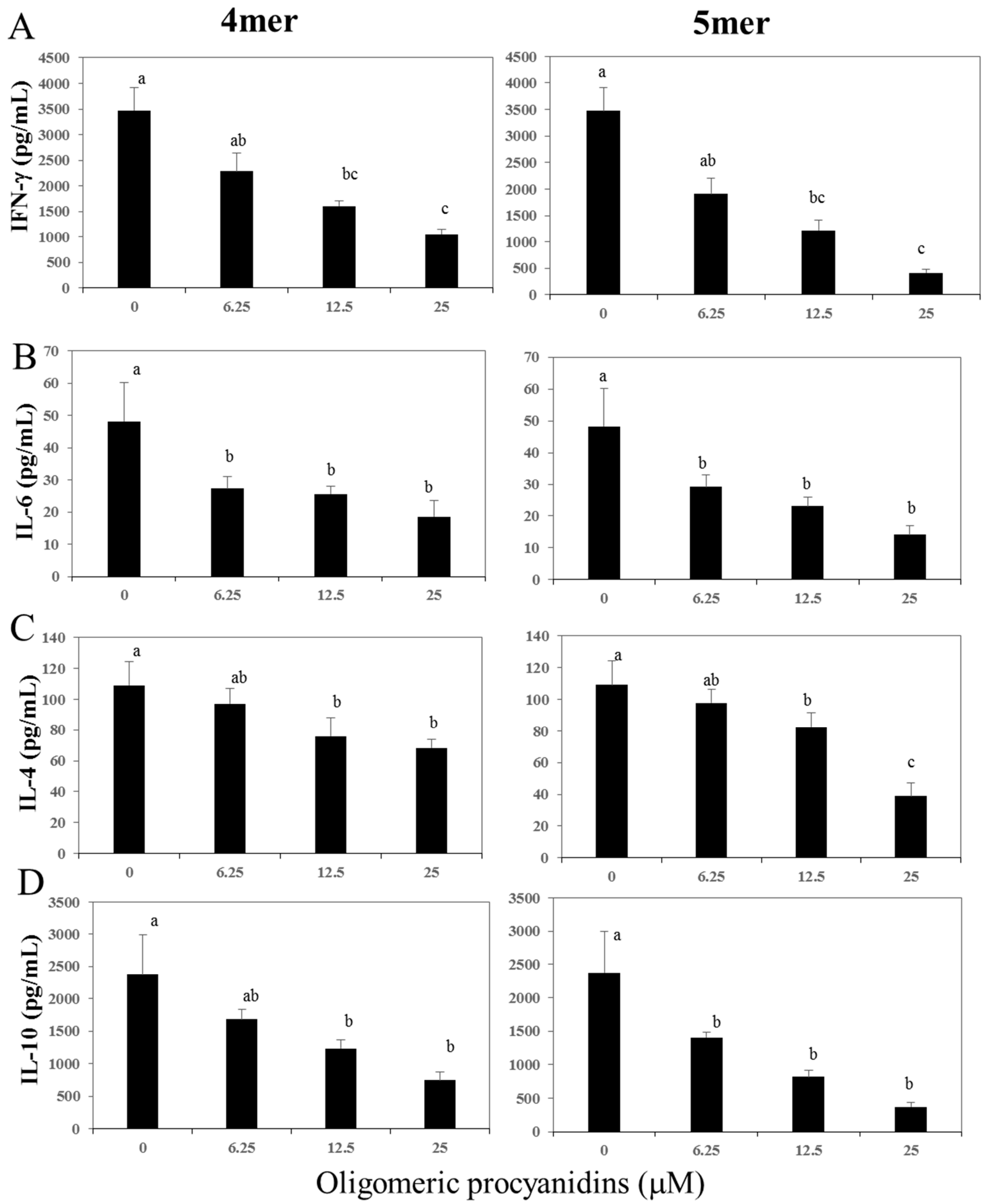

Figure S3. Dose-dependent effects of oligomeric procyanidins on the production of the effector cytokines, interferon (IFN)-γ (**A**); interleukin (IL)-6 (**B**); IL-4 (**C**); and IL-10 (**D**) by activated CD4^{+} T cells. Splenic CD4^{+} T cells were stimulated with an anti-CD3ε monoclonal antibody in the presence of tetrameric or pentameric procyanidins (0–25 μM). The data shown are the means ± SD from triplicate cultures. Values not sharing a common letter (a, b, c) differ significantly at $p < 0.05$ by the Tukey–Kramer multiple comparison test.

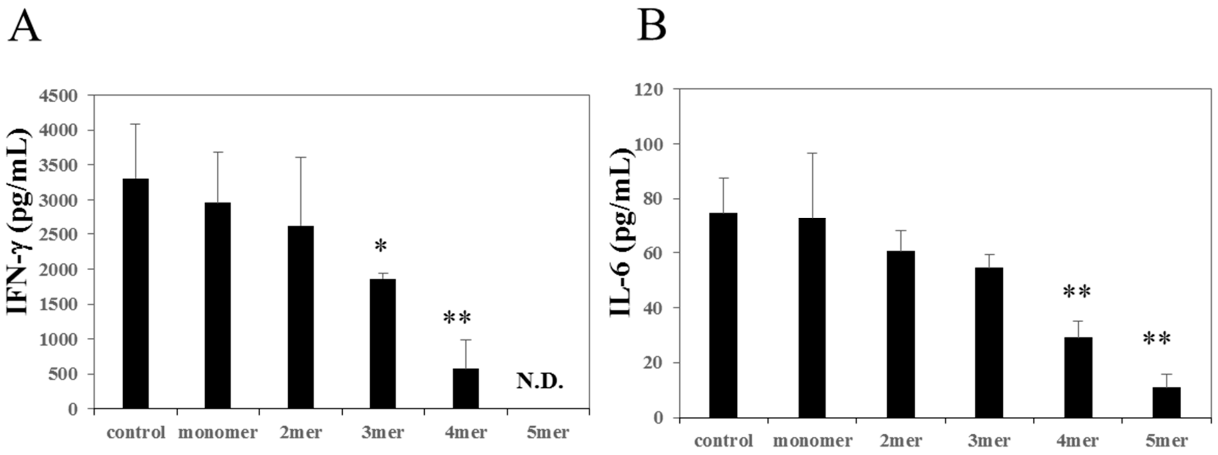

Figure S4. Effects of oligomeric procyanidins on the production of the effector cytokines, interferon (IFN)-γ (**A**) and interleukin (IL)-6 (**B**) of activated splenocytes from naïve DO11.10 mice. Pooled splenocytes were stimulated with 7.5 μM of ovalbumin (OVA) in the presence of oligomeric procyanidins (25 μM). The data shown are the means ± SD from triplicate cultures. Statistical comparisons were performed using analysis of variance with Dunnett's multiple comparison of means test. Significance is relative to a negative control (* $p < 0.05$, ** $p < 0.01$).
